# CRL4-DCAF8L1 Regulates BRCA1 and BARD1 Protein Stability

**DOI:** 10.7150/ijbs.57178

**Published:** 2022-01-24

**Authors:** Fei Liu, Qianying Han, Ting Zhang, Fen Chang, Jingcheng Deng, Xiaotian Huang, Weiping Wang, Yongjie Xu, Qin Li, Luzheng Xu, Bo Zhang, Wentong Li, Li Li, Yanrong Su, Yang Li, Genze Shao

**Affiliations:** 1 Department of Cell Biology, School of Basic Medical Sciences, Peking University Health Science Center, Beijing, 100191, China; 2 Department of Pathology, School of Basic Medical Sciences, Peking University Health Science Center, Beijing, 100191, China; 3 Department of Biochemsitry; School of Basic Medical Sciences, Peking University Health Science Center, Beijing, 100191, China; 4 Medical and Health Analysis Center, Peking University, Beijing, 100191, China; 5 Department of Pathology, Weifang Medical University, Shandong, 261041, China; 6 The Irma H. Russo, MD Breast Cancer Research Laboratory, Fox Chase Cancer Center-Temple University Health System, Philadelphia, PA, 19111, USA

**Keywords:** BRCA1, BARD1, DCAF8L1, ubiquitination, breast cancer, X chromosome inactivation

## Abstract

BRCA1 is frequently down-regulated in breast cancer, the underlying mechanism is unclear. Here we identified DCAF8L1, an X-linked gene product, as a DDB1-Cullin associated Factor (DCAF) for CUL4 E3 ligases to target BRCA1 and BARD1 for proteasomal degradation. Forced expression of DCAF8L1 caused reduction of BRCA1 and BARD1, and impaired DNA damage repair function, conferring increased sensitivity to irradiation and DNA damaging agents, as well as Olaparib, a PARPi anticancer drug; while depletion of DCAF8L1 restored BRCA1 and suppressed the growth of its xenograft tumors. Furthermore, the expression of DCAF8L1 was induced in human H9 ES cells during transition from primed to naïve state when Xi chromosome was reactivated. Aberrant expression of DCAF8L1 was observed in human breast fibroadenoma and breast cancer. These findings suggest that CRL4^DCAF8L1^ is an important E3 ligase that may participate in the development of breast cancer, probably through regulating the stability of BRCA1 and BARD1 tumor suppressor, linking BRCA1 and X chromosome inactivation to breast carcinogenesis.

## Introduction

The development of breast cancer is a multiple-step process associated with diverse events including activation of oncogenes and loss of tumor suppressors, among which the breast cancer susceptibility protein 1 (BRCA1) is considered to be one of the most susceptible protein 1, 2. Germline mutation of BRCA1 is very frequent in inherited breast cancer and predisposes women to breast cancer with high lifetime risk 3; In addition, down-regulation of BRCA1 is also observed in sporadic breast cancer. Decreased BRCA1 protein level is reported in 30-60% breast cancer tissues 4, 5. These findings suggested that BRCA1 might also play a role in the development of sporadic breast cancer, and loss of BRCA1 function due to insufficient BRCA1 protein could be one of the most important causes for the disease.

BRCA1 plays critical roles in DNA damage repair 6, 7, cell cycle and checkpoint control 8, 9. Each of these functions is essential for genome integrity and disruption is thought to promote cellular transformation and contribute to carcinogenesis 10, 11. In addition, BRCA1 was demonstrated to play an essential role in the regulation of mammary stem cell differentiation, particularly the differentiation of luminal epithelial lineage 12. Therefore, defect in DNA damage repair and impairment of mammary stem cell differentiation could be two plausible mechanisms for those breast cancers with loss of or insufficient BRCA1.

Protein homeostasis is important for the maintenance of cellular biological functions. It has been demonstrated that insufficiency of BRCA1 could affect some of, if not all, cellular functions of BRCA1. For example, heterozygous BRCA1 mutant cells displayed haploinsufficiency in replication fork repair, while other aspects of BRCA1 functions such as homologous recombination repair was not significantly affected 7, 13, 14. These studies suggest that the minimum amount of protein required for each disparate function of BRCA1 could be different, and insufficiency would also lead to genomic instability and cancer.

The cellular protein level of BRCA1 is tightly regulated by multiple mechanisms, among which the ubiquitin proteasome system (UPS) is considered to be the most important one. Many E3 ligases including SCF^FBXO44^, HERC2 and HUWE1 were reported to mediate the ubiquitination and proteolytic degradation of BRCA1 through UPS 15-17, playing an important role in regulating BRCA1 protein homeostasis. In addition, BRCA1 Associated RING Domain 1 (BARD1) can heterodimer and stabilize BRCA1 18, 19 , which is an alternative mechanism to control BRCA1 protein level. More importantly, the reciprocal regulation of protein quantity and interdependent function of BARD1 and BRCA1 were further demonstrated in conditional *Bard1*, *Brca1* or *Bard1/Brca1* double knockout mice 20. Despite these findings, the mechanism for the regulation of BRCA1 protein stability has not yet been fully elucidated. Since inhibition of UPS pathway using MG-132 can cause dramatic accumulation of cellular BRCA1, it is possible that UPS could be a major pathway for BRCA1 degradation, and other yet unidentified E3 ligases that are responsible for degrading BRCA1 might exist and play a crucial role in a subset of breast cancers 21. Indeed, many E3 ligases including CUL4A were seen overexpressed in breast cancers 22-24. However, it is currently unclear whether there is any linkage between these E3 ligases and BRCA1 in breast cancer tumorigenesis.

Here we report the identification of DDB1 and CUL4-associated factor 8-like protein 1(DCAF8L1), a novel DCAF protein which is encoded by an X-linked gene, plays crucial role in targeting BRCA1/BARD1 for proteasomal degradation, and might be involved in the development of breast cancer.

## Material and Methods

### Plasmids

The pcDNA3.1-Flag-HA-DCAF8L1 and pcDNA3.1-His-V5-CUL4A were generated by a polymerase chain reaction-based subcloning strategy using cDNA of MCF7 cells as template; pCMV-Tag2A-DCAF8L1, pEGFP-C2-DCAF8L1 and TG006-FH-DCAF8L1 were subcloned using pcDNA3.1-Flag-HA-DCAF8L1 as templates; pFast-Bac-GST-His-BARD1-119-777 (GST-His-ΔR-BARD1) and pFast Bac-Flag-BRCA1-His (Flag-BRCA1-His) were generated by subcloning strategy using pcDNA3.1-HA-BARD1 or BRCA1 as template. The prokaryotic expression vectors pGEX-6P-3-DCAF8L1-1-600, pGEX-6P-3-DCAF8L1-14-92, pGEX-6P-3-DCAF8L1-559-600, and pGEX-6P-3-DCAF8L1-519-600 were generated by PCR using pcDNA3.1-Flag-HA-DCAF8L1 as templates. The primer sequences used for plasmid construction were provided in the Table S1.

### Antibodies and Antibody generation

Rabbit anti-DCAF8L1 antibodies were either purchased from Novus Biologicals (NBP1-93435) or raised against the N terminus (14-92aa) or C terminus (519-600aa) of DCAF8L1 fused with GST and subsequently affinity-purified using agarose resins coupled with 4 different peptides corresponding to distinct regions of DCAF8L1, respectively. The peptides used for affinity purification of antibodies were D8L1-NP (C-TGDGGDTRDGGFLNDASTENQNTDSESSSED, synthesized), D8L1-CP (C-DEEELDESSSTSDTSEEEGQDR, synthesized), D8L1-519 (peptide corresponding to 519-600aa of DCAF8L1, purified from GST fused D8L1-519 expressed from E.coli). Briefly, each of these DCAF8L1 peptides was conjugated to agarose resins using a Sulfolink Coupling Agarose Resin Kit (20511ES25, YEASEN). The peptides-coupling agarose resins were then used to affinity purify antibodies from the rabbit sera either immunized with the N terminus (14-92aa) or C terminus (519-600aa) of DCAF8L1 fused with GST. Other antibodies used in this study were commercially purchased and their information was provided in Table S2 in the supplemental information.

### Cell culture and transfection

HEK293T, HeLa, HeLa S3, MCF10A, MCF10F, MCF7, LCC2, SUM-159, BT549, T47D, HCC1937, Hs578T, MDA-MB-231, MDA-MB-435 and HCC1954 cells were purchased from American Type Culture Collection (ATCC) and cultured as described on the ATCC official website. Conventional (primed) human ESC line H9 was maintained in Pluripotency Growth Master 1(PGM1)(CELLAPY, CA1007500) medium under 5% CO_2_, 37℃ and passaged by treatment with 1 mg/ml Collagenase type IV (Gibco) followed by sedimentation to remove single cells. For conversion of preexisting primed H9, we seeded about 2x10^5^ single cells on an MEF feeder layer in hESM supplemented with ROCK inhibitor Y-27632 (Stemgent, 10 mM). Two days later, medium was switched to 5i/L/A. Naive colonies were appeared and expanded polyclonally using Accutase (GIBCO) on an MEF feeder layer. Naive human pluripotent cells were maintained in serum-free N2B27-based media supplemented with 5i/L/A as described 25.

The Jet PRIME kits were used for transient transfection of plasmids or short interfering RNAs. The targeting sequences for relevant siRNAs or shRNA(Invitrogen) and production of Lentiviral particles were provided in the key resource table (Supplementary Table S2).

### Immunoprecipitation and pulldown assays

For immunoprecipitation, cells were harvested and lysed in NETN-400 buffer (20 mM Tris-HCl, pH 7.4, 400 mM NaCl, 0.1% Nonidet P-40, 0.5 mM EDTA, 1.5 mM MgCl_2_, 10% Glycerol) with protease inhibitors (p8340) and protein phosphatase inhibitors (PMSF) for 30 min on ice. The samples were centrifugated at 12000 rpm for 15 min, and the supernatants were diluted with NETN-0 (20 mM Tris-HCl, pH 7.4, 0.1% Nonidet P-40, 0.5 mM EDTA, 1.5 mM MgCl_2_, 10% Glycerol) to obtain final NaCl concentration of 150 mM. Protein G agaroses (Roche) were incubated with 1-2 µg antibodies at 4℃ with rocking for 2 h. The samples were then added (For anti-FLAG M2 agaroses, protein samples were directly added), and the incubation was continued for an additional 4 h. Agarose was then washed using the NETN-150 buffer. The bound proteins were eluted with 100 mM glycine, pH 2.5, and then neutralized by adding 1 M Tris-Cl, pH8.0 (1/10 volume of the elution buffer). Eluted proteins were separated by SDS-PAGE and blotted with the corresponding antibodies as indicated. For Ni-NTA pulldown, 48 h after transfection, cells were harvested and lysed in a phosphate/urea Buffer B (8 M Urea, 100 mM NaH_2_PO_4_, 10 mM Tris pH 8.0; 20 mM imidazole with freshly added 10 mM β-mercaptoethanol). The ubiquitinated proteins were precipitated with Ni-NTA Agarose (QIAGEN), followed by four washes with Buffer C (8 M Urea, 100 mM NaH_2_PO_4_, 10 mM Tris-HCl, pH 6.3). The precipitated proteins were eluted with Buffer E (8 M Urea, 100 mM NaH_2_PO_4_, 10 mM Tris-HCl, pH 4.0; 250 mM imidazole; 1 mM β-mercaptoethanol) and fractionated by 3%-8% SDS-PAGE, and analyzed by immunoblotting with indicated antibodies. For in vitro GST-pulldown, GST, GST-D8L1, and its truncations immobilized on glutathione-agarose beads were incubated with mitotic HEK293T cell lysates at 4℃ for 4 h respectively. The beads were washed extensively, and the proteins were eluted by adding 1 x SDS loading buffer, boiled and analyzed by immunoblotting.

### Protein expression and purification

For GST-D8L1 (GST-DCAF8L1) expression and purification, the plasmids were introduced into BL21 E.coli and GST fusion proteins were induced by IPTG (0.5-2 mM) treatment. Cells were then harvested and purified using Glutathione-Agarose. For GST-His-ΔR-BARD1 and Flag-BRCA1-His protein expression and purification, the Bac-to-Bac Baculovirus expression system (Invitrogen) was used. Briefly, pFastBac1-GST-His-ΔR-BARD1 or pFastBac1-Flag-BRCA1-His plasmid was constructed by introducing a GST and His-tagged BARD1 or Flag and His-tagged BRCA1 DNA fragment encoding amino acids 119-777 of BARD1 or full length of BRCA1 into pFastBac1, and transformed into DH10Bac Escherichia coli cells. Cells were selected with 50 µg/ml kanamycin, 7 µg/ml gentamicin, 10 µg/ml tetracycline, 100 µg/ml Bluo-gal, and 40 µg/ml IPTG. White colonies were confirmed by PCR and used for isolation of recombinant GST-His-ΔR-BARD1 or Flag-BRCA1-His Bacomid DNA. SF9 cells were transfected with GST-His-ΔR-BARD1 or Flag-BRCA1-His Bacomid DNA using Cellfectin (10362-100, Invitrogen) and baculovirus particles were generated and expanded. SF9 cells were infected with P2 stock baculovirus of GST-His-ΔR-BARD1 or Flag-BRCA1-His for 72 h before harvesting and lysing with SF9 lysis buffer (50 mM Hepes pH 8.0, 120 mM NaCl, 0.5% NP-40, 1 mM PMSF, proteinase inhibitor cocktails) for 40 min. The whole lysates were centrifuged at 12,000 rpm for 30 min. The recombinant proteins His-GST-BARD1-119-777 or Flag-BRCA1-His were first purified by Ni-NTA agarose gels and the eluates were subjected to a second purification, using either with glutathione Sepharose 4B (Amersham bioscience) or anti-Flag M2 agarose gels. The recombinant BARD1 and BRCA1 proteins were eluted with GST elution buffer (100 mM Tris-HCl, pH 8.0, 15 mg glutathione) or NETN-150 buffer containing Flag-peptides, respectively. For purification of CRL4^D8L1^ complex: Hela S3 cells were transfected with Flag-HA-D8L1 plasmid. Twenty-four hours later cells were reseeded for isolation of stable colonies expressing D8L1. Stable colonies were cultured and expanded. Cells were harvested and lysed in NETN-400 buffer (20 mM Tris-HCl, pH 7.4, 400 mM NaCl, 0.1% Nonidet P-40, 0.5 mM EDTA, 1.5 mM MgCl_2_, 10% Glycerol) with protease inhibitors and protein phosphatase inhibitors, for 30 min on ice. The samples were centrifugated at 12000 rpm for 15 min, and the supernatants were diluted with the same buffer without NaCl (20 mM Tris-HCl, pH 7.4, 0.1% Nonidet P-40, 0.5 mM EDTA, 1.5 mM MgCl_2_, 10% Glycerol) to obtain final NaCl concentration of 150 mM (NETN-150: 20 mM Tris-HCl, pH 7.4, 150 mM NaCl, 0.1% Nonidet P-40, 0.5 mM EDTA, 1.5 mM MgCl_2_, 10% Glycerol). The samples were then cleared by centrifugation and incubated with the appropriate antibodies at 4℃ with rocking for 2 h. Anti-FLAG M2 agarose gels were then added, and the incubation was continued for an additional 4h. Agarose gels were then washed three times using the NETN-150 buffer. The bound proteins were eluted with Flag-peptide. The Flag-peptide eluted proteins were diluted with 0.01 mM PBS and subjected to a second affinity purification using anti-HA agarose gels and eluted with HA peptide. The HA peptide eluted proteins were separated on SDS-PAGE and stained with silver, or aliquoted and stored at -80℃.

### Immunofluorescence microscopy and RNA FISH (Fluorescence in situ hybridization)

Cells grown on coverslips were fixed in 4% paraformaldehyde at room temperature for 10 min, washed three times with PBS, permeabilized in PBS supplemented with 0.4% Triton X-100, blocked with 10% goat serum at 37℃ for 1 h, incubated at 4℃ with the primary antibodies overnight, washed extensively and probed with the FITC- and Rhodamine red-conjugated goat anti-rabbit or anti-mouse IgG (Jackson Immuno Research Laboratories, Inc.) and Donkey anti-Mouse IgG (H+L) Secondary Antibody/Alexa Fluor 647 (A31571, Life Technologies) at 37°C for 1 h. Coverslips were mounted with DAPI (Vector Laboratories).

For *XIST* RNA assay, we used Quasar® 570-labeled human* XIST* probe (SMF-2038-1 Biosearch Tecnologies) to examined *XIST* mRNA of cells followed standard procedure.

### *In vitro* ubiquitination assays

For *in vitro* ubiquitination assay, different combinations of 100 ng E1 (E-305 BostonBiochem), 100 ng E2 (UbcH5c, E2-627, Boston Biochem) and 50 ng E3 (CRL4^DCAF8L1^) were mixed with 100 ng recombinant GST-His-ΔR-BARD1 substrate in a 25 µl reaction buffer (2 μg HA-ubiquitin, 2 mM ATP, 5 mM MgCl_2_, 2 mM DTT, 50 mM Tris-HCl, pH 7.4). Reactions were carried out for 90 min at 30℃, terminated by boiling for 10 min in a protein loading buffer, fractionated by 3%-8% SDS-PAGE, and blotted with required antibodies.

### Immunohistochemistry with breast microtissue arrays

All breast tissue arrays were purchased from Alenabio (http://xian089380.11467.com/about.asp) (the previous website was www.alenabio.com, cat#.BR1002a). The protocol of immunohistochemistry staining was conducted according to manufacturer's instructions. Antigen retrieval was performed with citric acid buffer (Solarbio, C1032) at about 95℃ for 20 min. 3% hydrogen peroxide and 10% goat serum were used to prevent endogenous peroxidases and nonspecific binding of antibodies. The tissue sections were incubated with rabbit polyclonal anti-DCAF8L1 antibody (cat.NBP1-93435; Novus Biologicals) or mouse monoclonal anti-BRCA1 antibody (Invitrogen, MS110) overnight at 4℃. GTVision TM III Detection System/Mo&Rb (GTR GK500705) was used for detection.

### HR and NHEJ repair reporter assays

HR and NHEJ repair reporter assay were conducted as described 26, the U2OS/DR-GFP and U2OS /EJ5-GFP cell lines are gifts from Dr Yongliang Zhao at Beijing Institute of Genomics, Chinese Academy of Sciences. Cells were transfected with plasmid I-SceI (pCBASce) together with empty or the indicated plasmids. 48 h post transfection cells were harvested and GFP positive cells were analyzed by FACS. The repair efficiency was scored and normalized with control as the percentage of GFP-positive cells. To assay the impact of individual genes in DSB repair, prior to the transfection with pCBASce, cells were treated with the indicated plasmids or the corresponding siRNAs against each gene for 36 h.

### Metaphase spread Assay

HeLa cells were transfected with Flag-HA-control or Flag-HA-DCAF8L1 plasmids. Cells were then treated with 200 ng/ml Mitomycin C (MMC, Sigma, M4287) for 48 h and incubated with 0.05 mg/ml Colcemid (Sigma, C9754) for another 16 h. Metaphase spreads were prepared as described 27 and examined using a x100 oil immersion lens with Axio Scope A1 biomicroscope.

### Cell survival and sensitivity assays

All cells were seeded into triplicate 60-mm plates at 200 cells/plates. 24h after treatment, cells were γ-irradiated at the indicated doses using GSR-D1 137Cs gamma-irradiator (RPS Services Limited), or exposed to Doxorubicin, Etoposide or Olaparib. For γ-irradiation, cells were incubated at 37℃for 14 days, and then the colonies were stained with Sulforhodamine B (SRB) and counted. For etoposide treatment, MCF10A cells were infected with control or TG006-FH-D8L1 virus for 72 h, then transferred into 96-well plates, and treated with or without etoposide (ETO, 10 µM) for 6 h, the survival rates were then assessed by MTT assay. For Doxirubincin treatment, MCF10A cells were infected with control or DCAF8L1 with or without BRCA1 lentivirus for 72 h, then seeded into 96-well plates, the cells were treated with or without Doxorubicin (DOX, 0.5 µg/ml), the survival rates of cells were assessed by MTT assay. For Olaparib treatment, MCF10A cells were infected with control or DCAF8L1 with or without BRCA1 lentivirus for 72 h, then seeded into triplicate 60-mm plates at 200 cells/plates, and treated with or without Olaparib at the indicated doses, the medium was changed every four days, and cells were incubated at 37℃ for 14 days, then the clones were stained with Sulforhodamine B (SRB) and counted.

### Xenograft tumorigenesis assay

All animal experiments were performed under the approval of the Animal Care and Use Committee of Peking University. Approximately 4x10^6^ of HCC1954 cells that stably express DCAF8L1 shRNA (shD8L1-1) and a scramble shRNA (shCT) were mixed in Matrigel and injected directly into mammary fat pads (left side of each mouse) of 6-7 week-old female NOD/SCID nude mice (n =5 for each group), respectively. The primary tumor volume was monitored weekly. Tumor volumes were measured with calipers and calculated according to the following formula: tumor volume = (length × width^2^)/2. Six weeks after injection, all the animals were killed and dissected. The paraffinized sections were stained with hematoxylin and eosin (HE). The stained sections were photographed using a Leica microscope.

## Results

### DCAF8L1 regulates cellular protein level and stability of BRCA1 and BARD1

**Figure 1:**
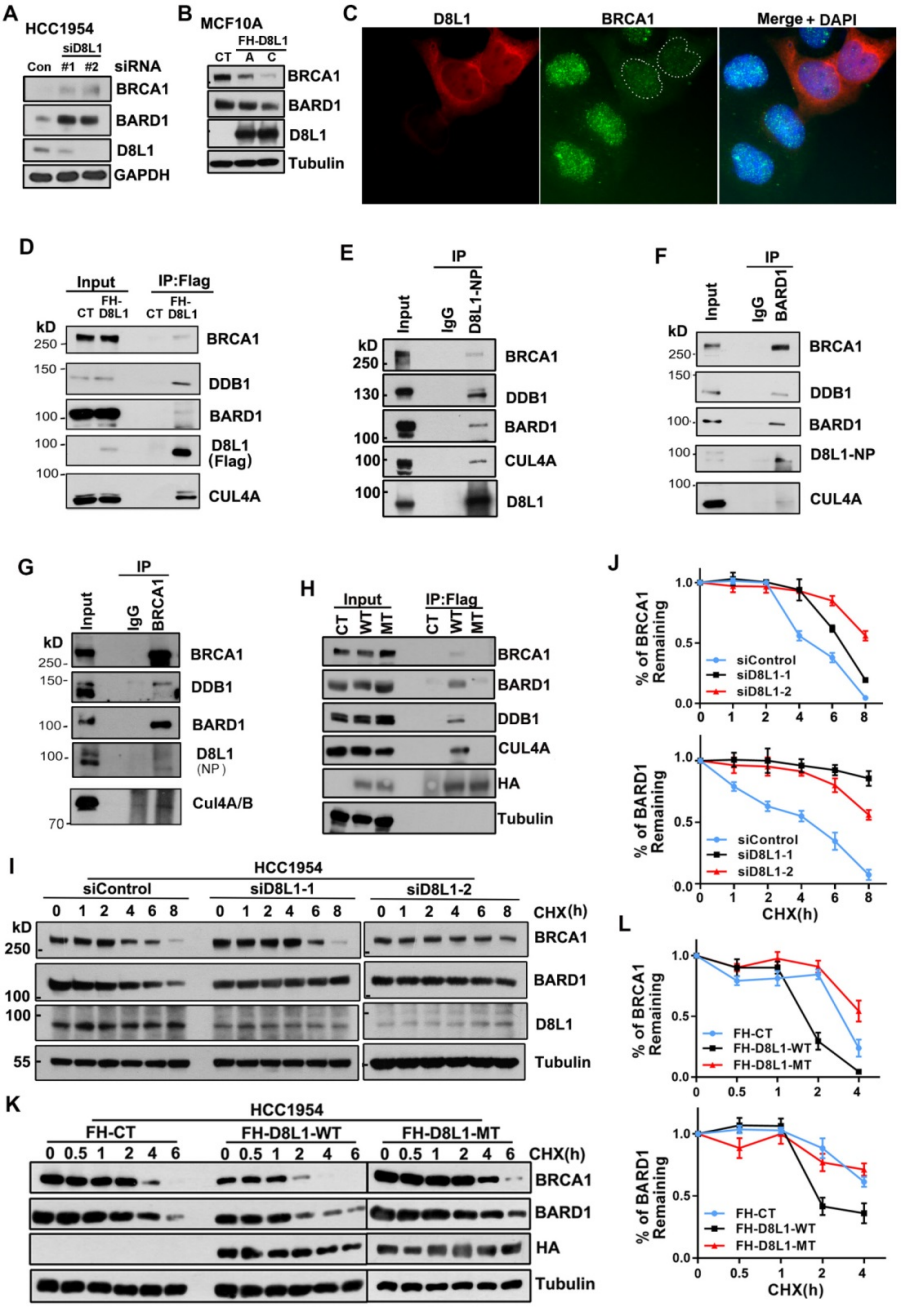
** DCAF8L1 interacts with BRCA1 and BARD1 and regulates their protein stability. (A)** Depletion of DCAF8L1 resulted in BRCA1 and BARD1 accumulation in HCC1954 cells. Cells were treated with two individual siRNAs against *DCAF8L1* or control siRNA, and cell lysates were subjected to immunoblotting using antibodies as indicated. **(B)** Overexpression of DCAF8L1 resulted in decreased BRCA1 and BARD1. MCF10A cells were infected with lenti-virus expressing Flag-HA tagged control (FH-CT) or DCAF8L1 (FH-D8L1). Two stable clones, clone A and C, were successfully obtained, BRCA1 and BARD1 proteins were examined by immunoblotting. **(C)** Forced expression of DCAF8L1 in MCF10A caused a decrease in BRCA1 protein. MCF10A cells were infected with lenti-virus expressing FH-D8L1 and IF was performed to detect BRCA1, white dashed line circles indicate cells expressing FH-D8L1. **(D)** Exogenous DCAF8L1 interacts with BRCA1 and BARD1. IP assays were performed on lysates from 293T cells expressing control or FH-D8L1 using anti-FLAG M2 agarose gel, and analyzed by immunoblotting with the indicated antibodies. **(E)** Endogenous DCAF8L1 interacts with BRCA1 and BARD1 in HCC1954 cells. IPs were performed using control IgG or antibody against DCAF8L1 (D8L1-519). Immunoblots were analyzed with another antibody against DCAF8L1 (D8L1-NP), or antibodies as indicated. **(F and G)** BARD1 and BRCA1 reciprocally interact with DCAF8L1 in HCC1954 cells. IPs were performed using control IgG or antibody against BARD1(F) or BRCA1(G), and analyzed with antibodies as indicated. **(H)** DCAF8L1 mutant (R317H, R365H, MT) does not form complex with CUL4A, DDB1 and BRCA1/BARD1. DCAF8L1 wild type (WT) and MT were pulled down and analyzed with the indicated antibodies. **(I and J)** Depletion of DCAF8L1 resulted in increased stability of endogenous BRCA1 and BARD1 proteins. HCC1954 cells were transfected with two individual siRNAs specifically against DCAF8L1. 66 h post-transfection, cells were treated with CHX (100 µg/ml) to block *de novo* protein synthesis and then harvested at the indicated times points after CHX treatment and protein levels were analyzed by immunoblotting (I). Quantification of the protein level of BRCA1 or BARD1 was plotted (J). Mean ± SD of three independent experiments. Student's t test. **(K and L)** Forced expression of DCAF8L1 accelerated degradation of BRCA1 and BARD1 proteins in HCC1954 cells. Cells were infected with lenti-virus expressing CT or FH-D8L1 and treated as in (I and J) and protein levels were analyzed by immunoblotting (K). Quantification of the protein level of BRCA1 or BARD1 was plotted (L). Mean ± SD of three independent experiments. Student's t test.

Previous studies showed that CUL4A was overexpressed in breast cancers 22-24, 28, suggesting that Cullin4-RING Ligase (CRL4) might play an important role in breast carcinogenesis. CRL4 is a large E3 ligase family with various substrates and the substrate specificity is determined by DCAFs 29, 30. In order to explore whether X-linked genes are involved in the regulation of BRCA1 through UPS, we searched genes on X-chromosome that can encode DCAFs for CRL4 and DCAF8L1 was identified as one of the candidates. *DCAF8L1* is localized on Xp21.3, and seems to be an intronless retrocopy of DCAF8 which is a multi-exon gene encoding a known DCAF associated with CRL4 31. As is shown (Figure S1A), *DCAF8L1* is predicted to encode a protein containing seven WD repeats. To study the function of DCAF8L1, we first generated a series of antibodies against distinct epitopes of DCAF8L1 (Figure S1A). The specificity of these antibodies for western blotting (WB), immunoprecipitation (IP) was assessed (Figure 1A and S1B). The D8L1-519 and D8L1-NP antibodies which were confirmed with no cross-reaction with DCAF8 or DCAF8L2 (Figure S1C) were used in this study.

To investigate whether DCAF8L1 plays a role in regulating BRCA1 or BARD1 protein, HCC1954 cells were depleted of DCAF8L1 with two individual siRNAs, and BRCA1 and BARD1 were examined. Knockdown of DCAF8L1 significantly increased the protein level of BRCA1 and BARD1 (Figure 1A); Consistently, stable overexpression of DCAF8L1 in MCF10A resulted in dramatic decrease of BRCA1/BARD1 protein level (Figure 1B). In addition, IF staining also showed that BRCA1 in MCF10A cells was depleted when DCAF8L1 was forcedly expressed (Figure 1C). These results suggest that DCAF8L1 is a negative regulator of BRCA1 and BARD1. To test this hypothesis, we first examined the interactions between DCAF8L1 and BRCA1/BARD1 using IP. DCAF8L1 was expressed in 293T cells and IP was performed. Immunoblotting showed that both BRCA1 and BARD1 were pulled down by ectopic Flag-HA-DCAF8L1 (Figure 1D, S2). Moreover, endogenous DCAF8L1 from HCC1954 cells could efficiently immunoprecipitate BRCA1 and BARD1 (Figure 1E). CUL4A/B and DDB1 were also detected in the IPs, suggesting that DCAF8L1, similar to DCAF8, could be an adaptor protein in the CRL4 E3 ligase complex (Figure 1D, E). Consistent with these results, DCAF8L1 was reciprocally detected in the IPs of either BARD1 or BRCA1 (Figure 1F, G). Interestingly, DCAF8L1 mutant (R317 and R365, two arginine residues that are required for the interaction with DDB1 were mutated to Histidine; MT) that doesn't bind DDB1 and CUL4A also did not efficiently interact with BARD1 or BRCA1 (Figure 1H). These results suggest DCAF8L1 could serve as a DCAF linking CRL4 with BRCA1 or BARD1.

CRL4 ubiquitin ligases are often associated with protein degradation through regulating the stability of their substrates 29. DCAF8L1 can interact with CUL4 and DDB1. Therefore, it could be one of the CRL4 E3 ligases. To determine whether CRL4^DCAF8L1^ is the E3 ligase responsible for BRCA1 and BARD1 degradation, we then examined the effect of DCAF8L1 on the stability of BRCA1 or BARD1, using cycloheximide (CHX) chase experiments in which protein synthesis was inhibited. As expected, depletion of DCAF8L1 in HCC1954 cells using two specific siRNAs resulted in significant stabilization of BRCA1 and BARD1 (Figure 1I, J), whereas forced expression of Flag-HA tagged, wild type DCAF8L1 (WT), but not its mutant, accelerated the degradation of both BRCA1 and BARD1 when compared with the control (Figure 1K, L). Similar result was obtained in MCF10A cells (Figure S3). These results indicate DCAF8L1 can regulate the cellular protein stability of BRCA1 and BARD1.

### DCAF8L1 interacts with BRCA1 and BARD1

**Figure 2:**
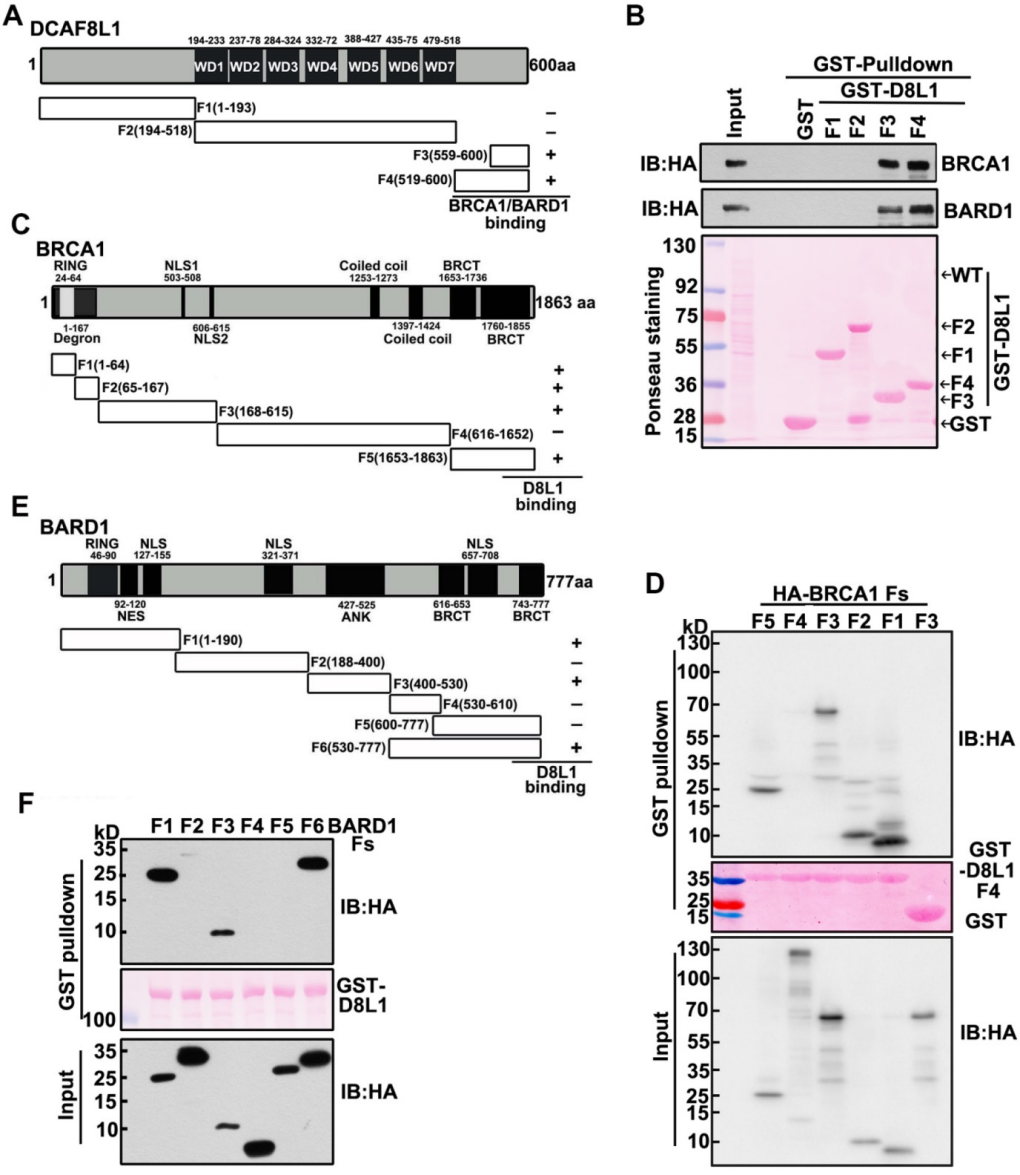
** Interaction Mapping of DCAF8L1 with BRCA1 or BARD1. (A)** Schematic diagram of mapping DCAF8L1 to BRCA1/BARD1. To map the region of DCAF8L1 required for association with BRCA1/BARD1, GST-tagged DCAF8L1 fragments were constructed. **(B)** Mapping of DCAF8L1 with BARD1 and BRCA1* in vitro*. GST-fused, truncated forms of DCAF8L1 proteins were purified from E.coli. Pull-down assays were performed using purified GST or GST fusion proteins with HEK293T cell lysates. The bound proteins were analyzed by SDS-PAGE and visualized by ponceau staining (bottom) and immunoblotted with BRCA1 or BARD1 antibodies. GST protein was used as negative control. **(C)** Schematic diagram of mapping BRCA1 to DCAF8L1. To map the region of BRCA1 required for association with the C terminus of DCAF8L1, a series of HA-tagged BRCA1 fragments were constructed. BRCA1/DCAF8L1 interaction was summarized in the right panel. **(D)** The C terminus of DCAF8L1 directly binds to BRCA1* in vitro*. GST-fused DCAF8L1 F4 protein was used for pull-down using HEK293T cell lysates expressing BRCA1 F1, F2, F3, F4 and F5. The experiment was performed as in (B). F3 was used as negative control. **(E)** Schematic diagram of mapping BARD1 to DCAF8L1. To map the region of BARD1 required for association with DCAF8L1, a series of HA-tagged BARD1 fragments were constructed. BARD1/DCAF8L1 interaction was summarized in the right panel. **(F)** DCAF8L1 directly binds to BARD1* in vitro*. The experiment was performed as in (B), except for using HA-tagged BARD1 fragments and GST-fused DCAF8L1 full length protein.

To further characterize the interaction between DCAF8L1 and BRCA1 or BARD1, we mapped the regions of DCAF8L1 required for interaction with BRCA1 or BARD1. Plasmids expressing a series of GST-tagged DCAF8L1 fragments (F1-F4) were constructed (Figure 2A), and their ability to directly associate with endogenous BRCA1/BARD1 was assessed in HEK293T cells by GST-pulldown assay. Immunoblotting showed that BARD1 and BRCA1 were detected in the pulldowns of F3 and F4, but not F1 or F2 of DCAF8L1 (Figure 2B). This result indicates that the C-terminus of DCAF8L1 comprising amino acids 519-600 is essential to mediate direct interaction with BARD1 and/or BRCA1.

Next, we mapped the regions of BRCA1 that can interact with the C-terminus of DCAF8L1. GST-D8L1-F4 was used for pulldown with cell lysates expressing BRCA1 fragments F1, F2, F3, F4 and F5 (Figure 2C). The result showed that all fragments except F4 could interact with the C terminus of DCAF8L1 (Figure 2D). These results indicate that the C-terminus of DCAF8L1 not only binds the degron, but also other domains of BRCA1. Similar strategy was employed for mapping of BARD1 with DCAF8L1. The result showed that full length GST-DCAF8L1 primarily interacts with the F1 (Ring domain, NES domain), F3 (ANK domain) and F6 (Hinge and BRCT domain) of BARD1, but not F2, F4 and F5 domains (Figure 2E, F). These results indicate that both the N and C termini of BARD1 are essential to mediate its interaction with DCAF8L1.

Taken together, these results indicate that DCAF8L1 forms complexes with CRL4 and directs its interaction with BRCA1 or BARD1, suggesting CRL4^DCAF8L1^ could be an E3 ligase for BRCA1 and BARD1.

### DCAF8L1 targets BARD1 and BRCA1 for ubiquitination and degradation

**Figure 3:**
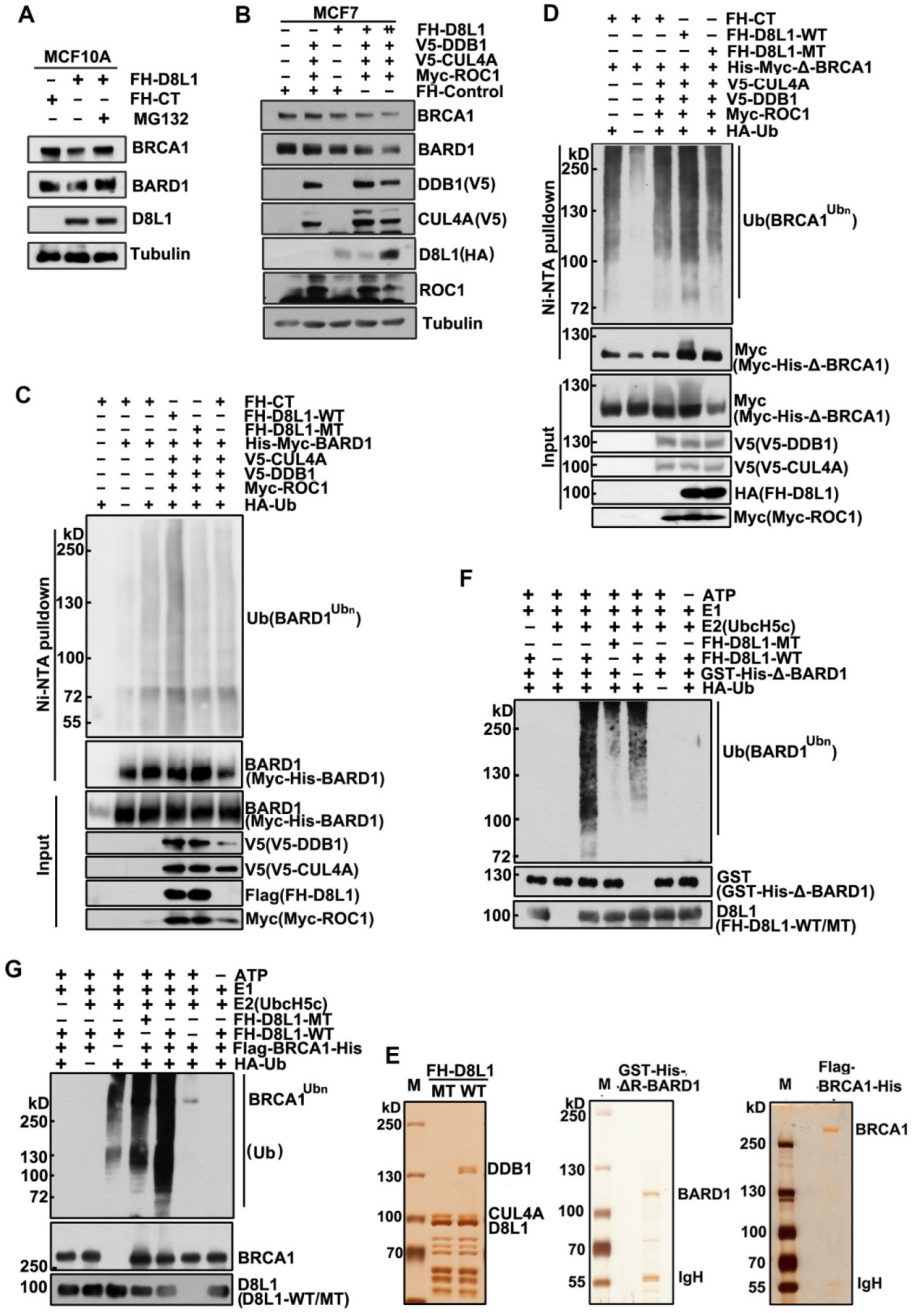
** CRL4^DCAF8L1^ ubiquitinates BARD1 and BRCA1 *in vivo* and *in vitro.* (A)** The cellular protein levels of BRCA1 and BARD1 were restored through treatment with the proteasome inhibitor MG-132. MCF10A cells were infected with lenti-virus expressing FH-CT or FH-D8L1. Sixty-eight hours post-infection cells were exposed to proteasome inhibitor MG-132 for another 4 h and analyzed by immunoblotting with the indicated antibodies. **(B)** Forced expression of CRL4^DCAF8L1^ caused decrease of BRCA1 and BARD1 in dose-dependent manner. MCF7 cells were transfected with the indicated plasmids and lysed 48 h later. Immunoblotting was performed using the indicated antibodies. **(C)** CRL4^DCAF8L1^ ubiquitinates BARD1 *in vivo*. HEK293T cells transfected with the indicated plasmids were treated with MG-132 for 6h before harvest, and Myc-His-tagged BARD1 protein was pulled down by Ni-NTA under denaturing condition. Immunoblotting was performed using the indicated antibodies. **(D)** CRL4^DCAF8L1^ ubiquitinates BRCA1 *in vivo*. Similar as in (C) except for using Myc-His-tagged BRCA1 plasmid without the Ring domain. **(E)** Silver staining of purified recombinant CRL4^DCAF8L1^ complex (left panel), GST-His-ΔR BARD1 (middle panel) and Flag-His-tagged BRCA1 protein (right panel). **(F)** *In vitro* ubiquitination assay. Recombinant GST-His-BARD1-119-777 (GST-His-ΔR-BARD1) proteins were incubated with E1, E2 (UbcH5c), ATP regenerating buffer, recombinant Flag-HA-D8L1 protein complexes and HA-ubiquitin or Myc-ubiquitin in a 25 µl reaction volume for 1.5 h at 30^o^C. The reaction mixtures were analyzed by immunoblotting with HA or BARD1 antibody. **(G)** CRL4^DCAF8L1^ promotes ubiquitination of BRCA1 *in vitro*. *In vitro* ubiquitination assay as in (F), using Flag-His-tagged BRCA1 protein as substrate.

Next, we investigated how DCAF8L1 regulates the stability of BRCA1 and BARD1. MCF10A cells infected with lentivirus expressing DCAF8L1 were treated with or without protease inhibitor MG-132, and cellular BRCA1 or BARD1 proteins were compared. Immunoblotting showed that both BRCA1 and BARD1 were decreased upon overexpression of DCAF8L1, but were rescued by MG-132 treatment (Figure 3A), suggesting they could be regulated by DCAF8L1 through ubiquitin-proteasome system. In support of this, expression of CRL4A E3 ligase complex with DCAF8L1 in MCF7 cells resulted in significant reduction of both BRCA1 and BARD1 in a dose-dependent manner (Figure 3B). Based on these findings, we therefore examined the role of DCAF8L1 in the ubiquitination of BARD1 or BRCA1 *in vivo* and* in vitro*. To simplify, we first used BARD1 as a substrate to test our hypothesis. HEK293T cells were transfected with His-tagged BARD1, HA-tagged Ubiquitin plasmids, and the CRL4 complex components plasmids (V5-tagged CUL4A and DDB1, and Myc-tagged ROC1) with or without DCAF8L1 wild type or its mutant. Immunoblotting analysis of the Ni-NTA pull-down showed that BARD1 was extensively ubiquitinated when CRL4A^DCAF8L1^ wild type, but not its mutant, was overexpressed (Figure 3C, and S4). Similar result was obtained when BRCA1 (BRCA1 truncate without the RING domain) was used as a substrate, the RING-deficient BRCA1 was robustly ubiquitinated upon forced expression of wild type, but not the mutant of DCAF8L1 complex (Figure 3D). These data suggest that CRL4A^DCAF8L1^ promotes the polyubiquitination of both BARD1 and BRCA1 *in vivo*.

To confirm the *in vivo* results, *in vitro* ubiquitination assays were conducted, using purified CRL4A-DCAF8L1 complex as E3 ligase and recombinant BARD1 or BRCA1 as substrate. DCAF8L1 mutant complex was used as control. Flag-HA-DCAF8L1 wild type or mutant was expressed in HEK293T cells and subsequently purified by affinity chromatography using an anti-FLAG-M2 agarose gel (Figure 3E, left panel). To avoid the interference of an intrinsic E3 ligase activity of BARD1, GST and His-tagged BARD1 without the N terminal 1-119 amino acids was expressed in Sf9 insect cells using baculoviruse- expression system, and then purified by Ni-NTA beads following affinity purification using GST-agarose column (Figure 3E, middle panel). Similarly, Flag- and His-tagged BRCA1 was also expressed in Sf9 insect system and purified (Figure 3E, right panel). *In vitro* ubiquitination assays demonstrated that the ubiquitination of BARD1 and BRCA1 were promoted in the presence of wild type, but not mutant DCAF8L1 protein complex (Figure 3F, lane 3, and Figure 3G, lane 5). Taken together, these data demonstrate that the CRL4A^DCAF8L1^ complex targets BRCA1 and BARD1 for ubiquitination.

### DCAF8L1 regulates cellular functions of BRCA1 in DNA damage repair

**Figure 4:**
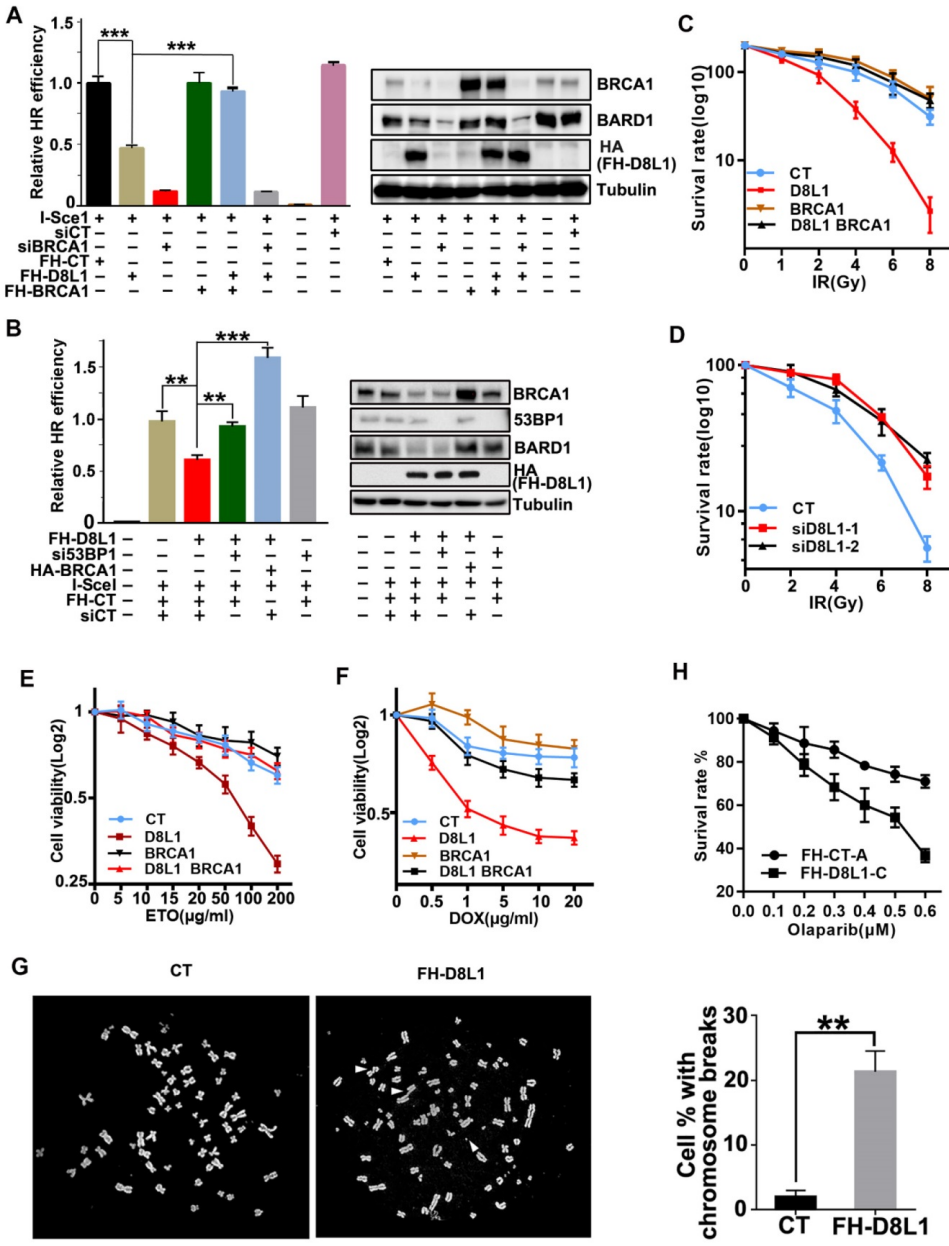
** Biological function of DCAF8L1 in DNA damage response. (A)** DCAF8L1 influences homologous recombination repair through negative regulation of BRCA1. U2OS-DR cells were transfected with or without the indicated plasmids or siRNA. 72 h post transfection cells were harvested and GFP positive cells were analyzed by FACS (left panel) and WB (right panel). *P<0.05, **P<0.01. **(B)** Depletion of 53BP1 restored homologous recombination efficiency in DCAF8L1 overexpressing cells. The experiment was performed as in (A). *P<0.05, **P<0.01. **(C)** Over-expression of DCAF8L1 sensitized MCF10A cells to IR. MCF10A cells were infected with control or DCAF8L1 together with or without lenti-virus expressing BRCA1 and subjected to different dosages of IR. Cell survival rate was calculated by counting the colonies formed two weeks after irradiation. **(D)** Depletion of DCAF8L1 confers IR resistance to T47D cells. DCAF8L1 was knocked down by two individual siRNAs in T47D cells. Cells were then treated as in (C). **(E)** Over-expression of DCAF8L1 sensitized cells to ETO. MCF10A cells were infected with control or DCAF8L1 together with or without lenti-virus expressing BRCA1 and exposed to various concentration of ETO. The survival rate of cells was assessed by MTT assay at the indicated time points. Data were presented as mean ± S.D. **(F)** Over-expression of DCAF8L1 sensitized cells to DOX. MCF10A cells were treated as in (E) except for DOX (0.5-20 μg/ml). The survival rate of cells was assessed by MTT as in (E). **(G)** A metaphase spread assay was carried out with Hela cells over-expressing DCAF8L1 or control as indicated. The broken chromosomes from over-expressing DCAF8L1 cells were marked with white arrows. *P<0.05, **P<0.01. **(H)** Over-expression of DCAF8L1 sensitized cells to Olaparib. MCF10A stable cell line over-expressing DCAF8L1 (FH-D8L1-C) or control (FH-CT-A) were treated with different dosage of Olaparib. Cell survival rate was calculated by counting the colonies formed two weeks post treatment.

To investigate the impact of DCAF8L1 on cellular function of BRCA1, we first examined whether BRCA1 dependent DNA damage repair is affected by DCAF8L1. As expected, overexpression of DCAF8L1 caused significant reduction of BRCA1 and decreased efficiency in homologous recombination repair, which was rescued by ectopic expression of BRCA1 (Figure 4A). More importantly, overexpression of DCAF8L1 didn't affect homologous recombination (HR) repair in cells depleted of BRCA1 by RNAi (Figure 4A). Previous studies demonstrated that BRCA1-dependent HR repair could be antagonized by 53BP1, a crucial factor promoting DNA damage repair choice towards Non-homologous end joining (NHEJ) repair pathway. Consistently, decreased HR repair efficiency caused by DCAF8L1 over-expression was restored when 53BP1 was depleted (Figure 4B). Overexpression of DCAF8L1 also mildly affected NHEJ repair (Figure S5). In line with an impaired HR repair function, cells overexpressing DCAF8L1 displayed increased IR sensitivity, which was rescued by BRCA1 re-expression (Figure 4C). Accordingly, depletion of DCAF8L1 conferred resistance to IR in T47D cells (Figure 4d). Similar results were obtained when cells were exposed to other DNA damaging agents such as etoposide (ETO) and doxorubicin (DOX). Forced expression of DCAF8L1 conferred cells increased sensitivity to etoposide and doxorubicin when compared with controls (Figure 4E, F). Compromised HR repair function arising from DCAF8L1 overexpression also led to genomic instability as indicated by increased chromosome aberrations (Figure 4G). Collectively, these results suggest that DCAF8L1 may participate in DNA damage repair through regulating BRCA1.

Previous studies have shown that BRCA1-deficient cells were sensitive to poly(ADP-ribose) polymerase inhibitors (PARPis), and this synthetic lethality depends on BRCA1-mediated HR function 32, 33. To investigate whether DACF8L1-mediated regulation of BRCA1 could also affect sensitivity to PARPi, MCF10A cell line stably overexpressing DCAF8L1 was generated and treated with Olaparib, one of the PARPis used for the treatment of BRCA1-deficient breast cancers in the clinics. As shown (Figure 4H), overexpression of DCAF8L1 resulted in significant reduction of cellular BRCA1 protein level and sensitized cells to Olaparib when compared with mock cells. Based on the fact that DCAF8L1 is overexpressed in many breast cancers, these findings might have important implications in cancer therapy.

### DCAF8L1 expression is induced in the differentiation and development of human stem cell

Human female cells contain two X chromosomes and one of them is subjected to inactivation (XCI). The establishment and maintenance of XCI is an epigenetic process, which plays an important role in mammalian development and stem cell differentiation. Loss of XCI can cause increased dosage of X-linked genes, which is thought to represent a key event in oncogenesis and stem cell development and differentiation 34, 35.

**Figure 5:**
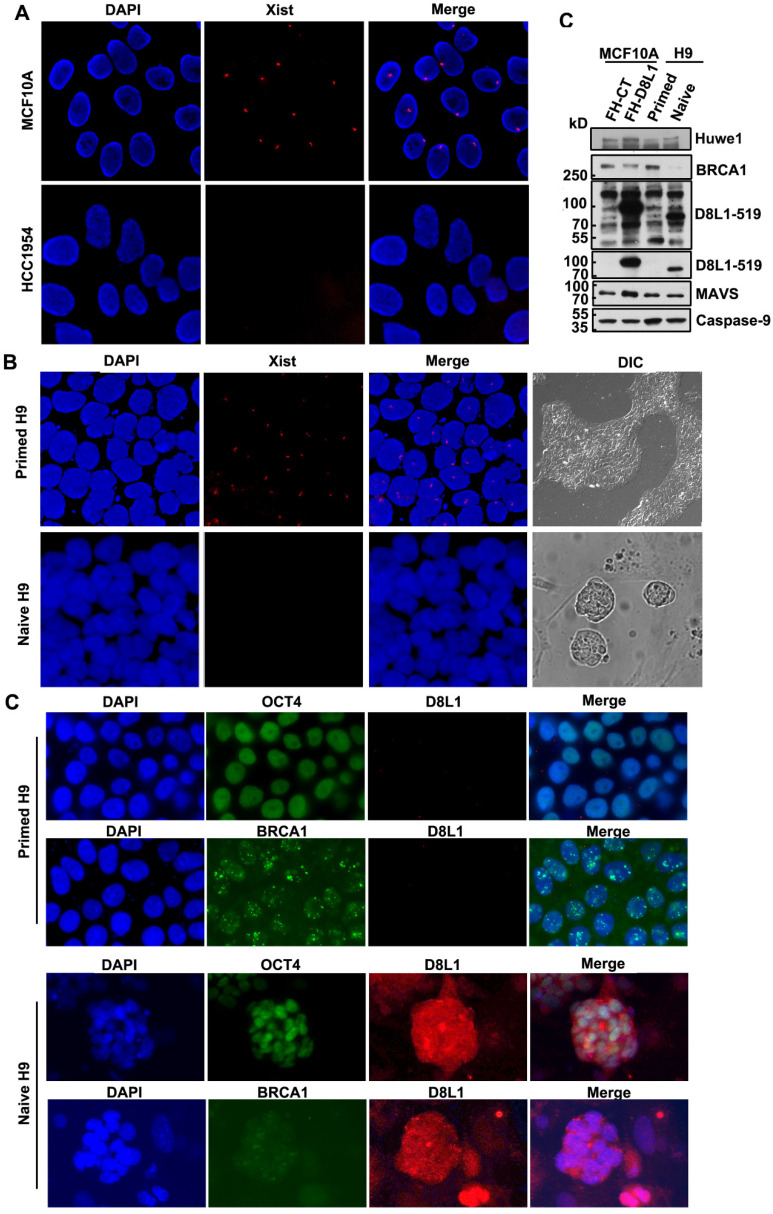
** Expression of DCAF8L1 is regulated by XCI status. (A)** The X chromosome inactivation status of MCF10A and HCC1954 cell lines. *XIST* RNA FISH was performed using immunofluorescence-labeled *XIST* RNA probe. **(B)** The Xi of primed hESC H9 cells were reactivated during transition to naive culture conditions. *XIST* RNA FISH was performed as in (A). **(C)** Representative IF images of DCAF8L1 or BRCA1 staining in primed or naive H9 cells. Cells were stained with antibodies against BRCA1(sc-6954), DCAF8L1(D8L1-519) or stem cell marker OCT4, respectively. **(D)** DCAF8L1 expression was induced in naive human ESC H9 cells. Primed H9 cells were induced to naive state and cells were harvested and lysed. Immunoblotting was performed using the indicated antibodies. MCF10A control or DCAF8L1-expressing stable cell lines were used as control.

*DCAF8L1* gene is an X-linked gene. To test whether a correlation between upregulation of DCAF8L1 and loss of XCI could be existed, we first examined status of XCI in MCF10A and HCC1954, two cell lines with differential expression of DCAF8L1, using *XIST* RNA FISH assay. As expected, low DCAF8L1-expressing MCF10A cells are *XIST* positive; whereas high DCAF8L1-expressing HCC1954 cells are *XIST* negative (Figure 5A). This result suggests that high expression of DCAF8L1 in HCC1954 cells could be a result from loss of XCI and reactivation of X chromosome (XCR).

To further confirm the influence of XCR on expression of DCAF8L1 and thus on BRCA1, we employed a primed human embryonic stem cell (hESC) line H9 and induced it to naive state. It is reported that the primed hESC cells could undergo reprogramming, and the Xi could be reactivated during the transition from primed to naïve state 36. The XCI status of primed H9 is XiXa, with one X chromosome inactivated, which is confirmed by *XIST* RNA FISH. Induction to naive state caused loss of XIST RNA and significant increased expression of DCAF8L1, as well as HUWE1, an E3 ligase encoded by an X-linked gene and negative regulator of BRCA1^16^, but had no significant effect on the expression of *MAVS* and *Caspase-9*, two genes on autosomal chromosomes (Figure 5B, D). Importantly, in consistence with a substrate as of DCAF8L1 and HUWE1, BRCA1 was shown dramatically decreased (Figure 5C, D). Since BRCA1 has been demonstrated to play critical roles in DNA damage repair and stem cell differentiation and development 12, 37, 38, this result suggests DCAF8L1 may play an important role in regulating stem cell differentiation, providing new insight in our understanding the mechanism of breast carcinogenesis.

### DCAF8L1 is involved in breast carcinogenesis

**Figure 6:**
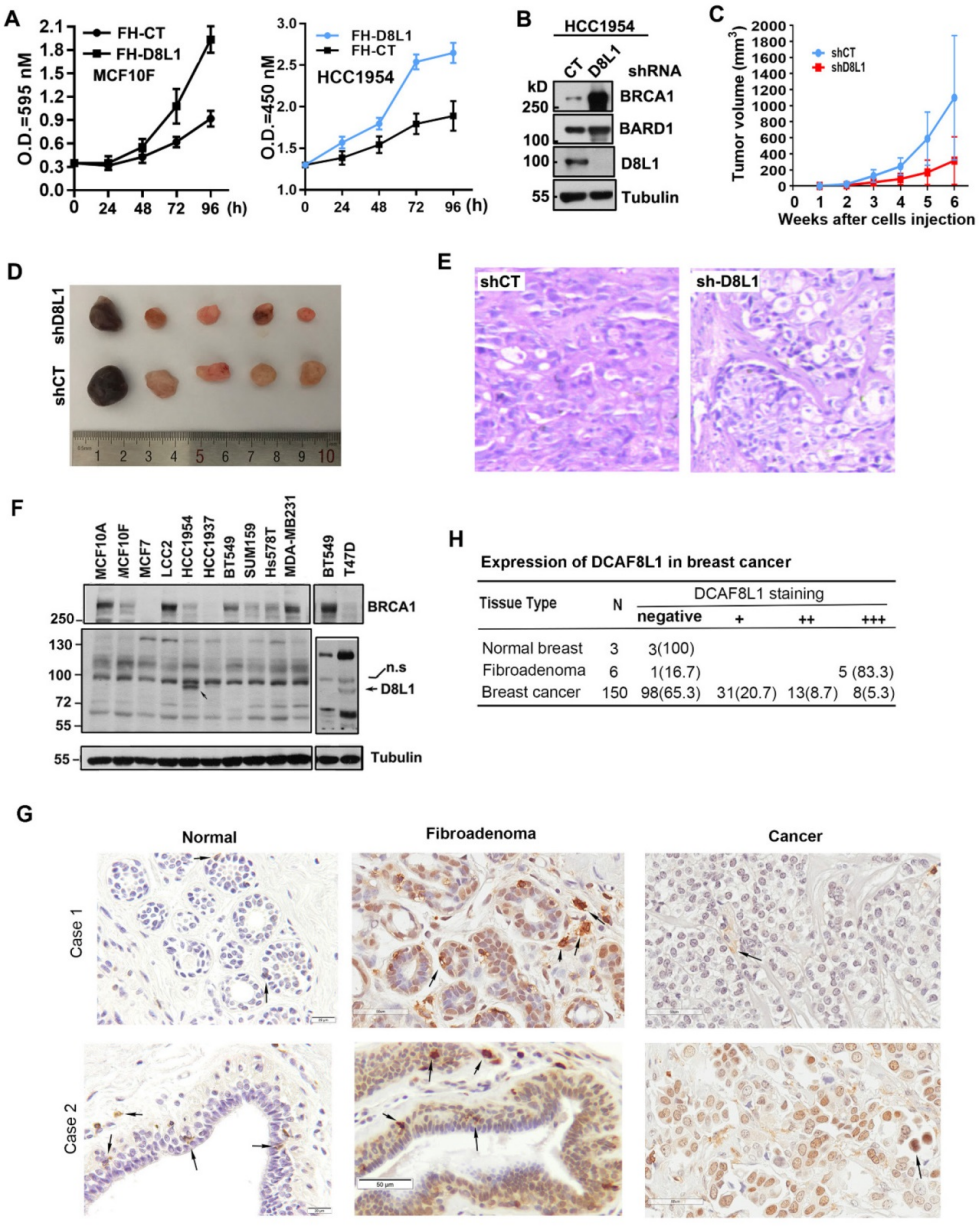
** DCAF8L1 is involved in breast cancer carcinogenesis. (A)** Over-expression of DCAF8L1 in MCF10F or HCC1954 promoted cell growth and proliferation. MCF10F cells (left panel) or HCC1954 (right panel) were infected with control or DCAF8L1 lentivirus. 72 h post infection cells were plated in 96-well plates. The survival rate of cells was assessed by MTT assay at the indicated time points. Data were represented as mean ± S.D. All of the assays were performed in triplicate. **(B)** Confirmation of knockdown of DCAF8L1 in HCC1954 cells by shRNA. shRNA-1 was used to knock down DCAF8L1, immunoblotting was performed with the indicated antibodies. **(C) (D)** and **(E)** Depletion of DCAF8L1 suppressed the growth of HCC1954 xenografts in nude mice. Cells infected with lenti-virus expressing control or DCAF8L1 shRNA were implanted into the mammary fad pad of nude mice as described in Materials and Methods. Tumor growth was monitored by measuring the tumor sizes at different time points (C). At the end of experiment, tumors were collected from the mice and measured. Representative tumor images were shown(D). HCC1954 xenograft tumors were verified by HE staining (E). **(F)** Expression of DCAF8L1 in immortalized breast epithelial and cancer cell lines. D8L1-NP, BRCA1 (sc6954) antibodies were used for the detection of DCAF8L1, BRCA1 by immunoblotting, respectively. Tubulin was used as loading control. n.s indicate a nonspecific band of DCAF8L1. **(G)** Expression of DCAF8L1 in human breast cancer and normal breast tissues. Representative IHC images of DCAF8L1 staining in normal breast, breast fibroadenomas and breast cancers are shown, using DCAF8L1 antibody (cat. NBP1-93435, Novus Biologicals). Arrows, cells with strong DCAF8L1 staining. Magnification, 40 ×; scale bars: 20 μm or 50 μm. **(H)** DCAF8L1 expression in human normal breast, breast fibroadenoma and breast cancers tissues. Left, normal breast; middle, fibroadenomas; right, breast cancers. Upper panel, case 1; lower panel, case 2.

To validate whether the oncogenic potential of DCAF8L1 is important in breast tumor progression, we first examined whether overexpression of DCAF8L1 could promote cell proliferation. Forced expression of DCAF8L1 in MCF10F or HCC1954 cells promotes cell proliferation (Figure 6A); next, we investigated whether depletion of DCAF8L1 in HCC1954 cells could affect tumor growth *in vivo*. DCAF8L1 in HCC1954 cells were depleted by shRNA lentivirus infection. The efficiency of shRNA to knock down DCAF8L1 was confirmed by Western blotting (Figure 6B). DCAF8L1 depleted cells or the vector control cells were subcutaneously injected (4 × 10^6^ cells) to mammary fat pad of female SCID mice, tumor latency and growth were recorded. All mice injected with vector control cells developed larger tumors as confirmed by IHC, whereas mice injected with the DCAF8L1 shRNA HCC1954 cells developed smaller tumors even after a prolonged time period (Figure 6C-E). This finding suggests DCAF8L1 may play an important role in tumor development.

Next, the expression of DCAF8L1 was examined in breast cancer cell lines and tissues. Immunoblotting showed that DCAF8L1 was expressed mainly in HCC1954 and T47D, two breast cancer cell lines with luminal epithelial characteristics (Figure 6F). The expression of DCAF8L1 was also examined by IHC with a commercial antibody (cat. NBP1-93435, Novus Biologicals), using breast tissue microarray containing breast fibroadenomas, invasive ductal carcinoma (IDC) tissues and normal breast tissues. The IHC analysis was carried out by pathologists according to the criteria shown in supplementary Table S3. The immunostaining pattern was divided into high, low and negative, according to the score based on total staining intensity and proportion of positive cells. The representative images were shown (Figure 6G). DCAF8L1 expression is negative in ducts and terminal duct lobular units (TDLUs) of normal breast tissues (Figure 6G, right panel), and is significantly upregulated in breast fibroadenomas (Figure 6G, middle panel). In contrast to fibroadenomas, most of the breast cancers do not express DCAF8L1; Of all the 150 cases of breast invasive ductal carcinoma tissues examined, 98 (65.3%) are negative, and 31 are weak in DCAF8L1 staining; only 8(5.3%) display high expression with nuclear staining of DCAF8L1(Figure 6H). Interestingly, strong cytoplasmic DCAF8L1 staining was also observed in a few yet-unidentified type of mammary cells in both normal breast, breast fibroadenomas and breast cancer tissues (probably tissue macrophages or tumor infiltrating lymphocytes, TILs) (arrows in Figure 6G). Immunofluorescence co-staining showed that BRCA1 is absent or significantly reduced in the cells overexpressing DCAF8L1 (Figure S6). Collectively, these results indicate that DCAF8L1 is aberrantly expressed in breast cancer cell lines and tissues, suggesting that alteration of DCAF8L1 might be associated with breast cancer progression and development.

## Discussion

In this study we demonstrated that CRL4^DCAF8L1^ is a novel E3 ligase that negatively regulates BRCA1 and BARD1, and plays an important role in BRCA1 dependent DNA damage repair. DCAF8L1 is aberrantly expressed in breast cancers. In addition, DCAF8L1 expression is induced in human ESC cell line H9 during transition from primed to naïve state. Our study provides a new insight into the mechanism of breast carcinogenesis. Since DCAF8L1 is an X-linked gene, and many breast cancers display X chromosome misbehavior including X chromosome duplication and loss of XCI, our study may have important implications in understanding the relationship between BRCA1, X chromosome instability and breast tumorigenesis.

Loss of *Barr* body is a common phenotype observed in many breast cancer cells^39^, ^40^. *Barr* body is a heterochromatin structure containing the Xi in female somatic cells. Female somatic cells usually undergo XCI to achieve similar X-linked gene dosage to male 41, 42. The loss of *Barr* bodies together with the fact that incidence of female breast cancer is significantly higher (approximately 100 fold) than that of male 43, suggest that the mechanisms for X chromosome silencing might be compromised in breast cancer cells, and changes of X-linked genes due to X chromosome abnormality might play an important role in breast carcinogenesis.

A consequence of X chromosome abnormality is the change of X-linked gene expression, including upregulation of oncogenes and/or down-regulation of tumor suppressor genes 44. Upregulation of global X chromosome genes or genes in certain region of X chromosome (eg. Xp22) was reported in breast cancer cells with X chromosome abnormality 44. Presumably, dysregulation of X chromosome genes could in turn exacerbate instability of genome including X chromosome itself, leading to tumor development and progression. Consistent with this notion, we found that X-linked gene DCAF8L1 is upregulated in some of the breast cancers; in addition, loss of Xi was also observed in DCAF8L1-overexpressing cell line HCC1954 (Figure 5A). These data suggest that misbehavior of X chromosome might drive the expression of DCAF8L1, leading to loss or insufficiency of BRCA1, which might in turn promote X chromosome instability. So far the mechanism for how X chromosome instability is initiated is unknown, oncogenic stresses, dysregulation of hormonal signaling and epigenetic reprograming might play an important role in the process.

Apart from its negative effect on genome integrity, overexpression of DCAF8L1 might also play a crucial role in promoting cell growth, proliferation or differentiation of mammary epithelial cells including stem/progenitor cells. In this study, we observed that DCAF8L1 is frequently overexpressed in breast fibroadenomas, benign neoplasms associated with breast cancer 45; it is also expressed in a small proportion of breast cancers; furthermore, expression of DCAF8L1 is induced in naïve hESC H9 cells in which BRCA1 is also downregulated. Consistently, previous studies have suggested a role of BRCA1 in regulating cell proliferation, mammary stem/progenitor cell development and differentiation 38, 46, 47. Taken together, these findings suggest that DCAF8L1 could promote cancer cell proliferation and regulate mammary stem/progenitor cell development and differentiation, probably through a mechanism dependent on its negative regulation of BRCA1 protein.

The *DCAF8* gene family in human genome contains three genes, the *DCAF8*, *DCAF8L1* and* DCAF8L2*. *DCAF8L1* and *DCAF8L2* are evolutionarily emerged only in primates, and they are intronless and considered as retro-copies of DCAF8. Unlike DCAF8 which is widely expressed in most of the human tissues and cells, DCAF8L1 is restrictedly expressed in testis and fallopian tubes (human protein atlas, https://www.proteinatlas.org/), suggesting it might have a role in human reproductive system. In this study we found DCAF8L1 is expressed in most of the human fibroadenomas and in a small proportion of the breast cancer cell lines (HCC1954 and T47D) and tissues (21% with medium to high expression). Interestingly, the expression of DCAF8L1 is also observed in a few of yet-unidentified cells (seems to be breast tissue macrophages or breast tumor infiltrating lymphocytes, TILs) in both fibroadenomas and invasive breast cancers (Figure 6G, arrows). This type of high-DCAF8L1 expressing cells was also found in many of the breast cancers, in particular the triple negative breast cancers (40-50%). These results suggest that DCAF8L1 might participate in breast carcinogenesis through influencing both mammary epithelial cells and the cells in the mammary stroma or tumor microenvironments.

Previous study has shown that CRL4^DCAF8^ can function as E3 ligase to ubiquitinate histone H3K79 and promotes H3K9 methylation, thus repressing transcription of fetal and cell-cycle genes in postnatal mouse liver 31. It is unclear whether DCAF8L1 has similar function as DCAF8; however, due to their high homology in protein sequence, it is possible that some of their functionalities might be overlapped. Indeed, our recent study showed that DCAF8 can interact and ubiquitinate BRCA1, playing a role in regulating BRCA1 stability. Unlike DCAF8L1, DCAF8 does not bind to BARD1 (manuscript in preparation). These findings suggest that the functionality of DCAF8 is evolutionarily diverged when the paralogues of DCAF8 such as DCAF8L1 and DCAF8L2 emerges in the primates. One intrinsic difficulty for DCAF8L1 research is that human DCAF8L1 does not have orthologues in mice, where most of experiments are performed. Future study will be needed for our understanding the precise role of each member of these DCAF8s and how they are coordinated to contribute to breast carcinogenesis.

Taken together, we identified DCAF8L1 as a novel E3 ligase to target BRCA1 and BARD1 for ubiquitination and degradation. Since BRCA1-deficient breast cancer is sensitive to PARPi^33^, ^48^, future exploration using PARPi for the treatment of these cancers with high DCAF8L1 expression will have important implications.

## Supplementary Material

Supplementary figures and tables.
