# Supplementary material for: CRL4-DCAF8L1 Regulates BRCA1 and BARD1 Protein Stability
Source: Int J Biol Sci. 2022 Jan 24;18(4):1434–50. doi: 10.7150/ijbs.57178 (PMC8898372; doi:10.7150/ijbs.57178)
Supplement: Supplementary file 1 — Supplementary figures and tables. [file ijbsv18p1434s1.pdf]

1. Supplementary Figures

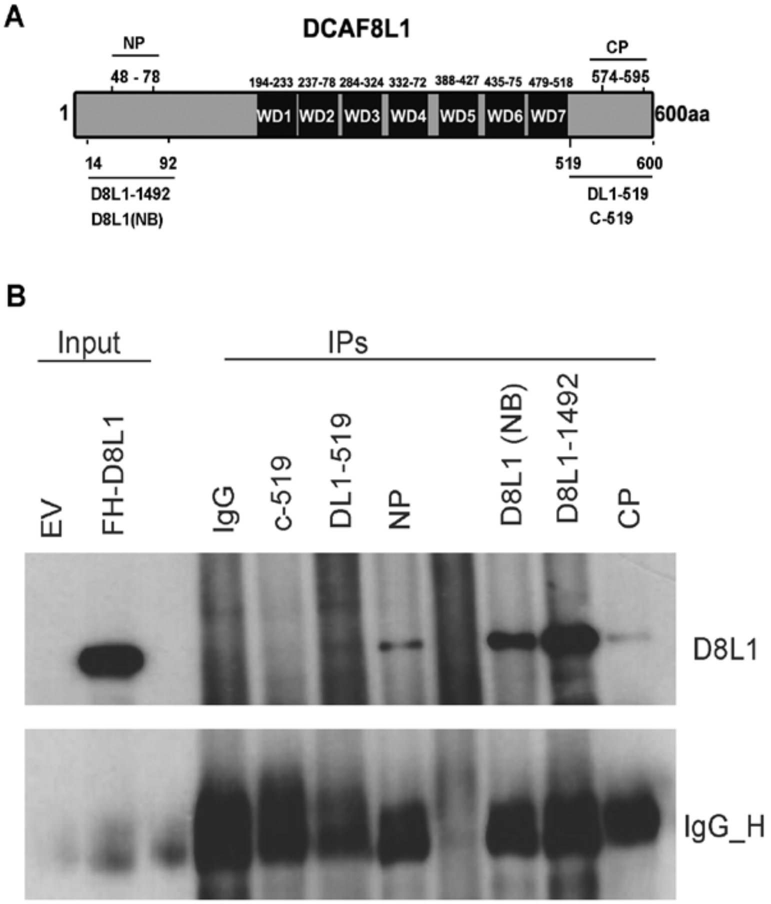

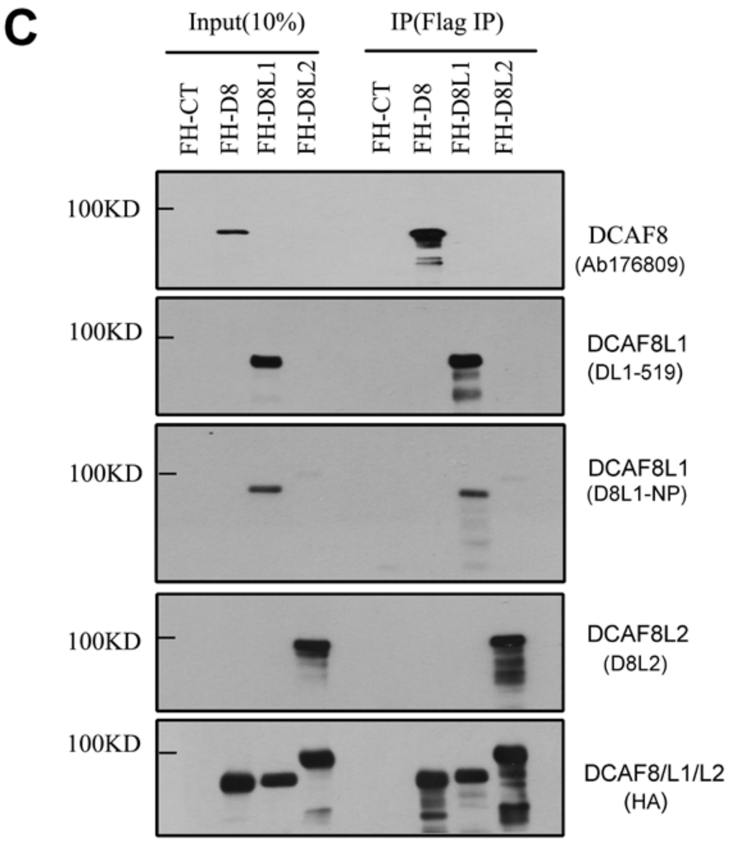

Fig.S1 Confirmation and Verification of specificity and application of antibodies against DCAF8L1.

(A) Schematic representation of the structure of DCAF8L1 protein and the corresponding regions for antibody generation and purification. Seven WD40 repeat domains and the regions corresponding to the peptides used for affinity purification of DCAF8L1 antibodies were indicated. NP, N-terminal peptide; CP, C-terminal peptide; D8L1-1492, Region14-92; D8L1(NB), DCAF8L1 antibody from Novus Biologicals (NBP1-93435)

(B) Application of DCAF8L1 antibodies for Immunoprecipitation (IP). Immunoprecipitation assays were performed on Flag-HA–DCAF8L1 (FH-D8L1) stable cell line with control IgG or indicated antibodies against D8L1 and analyzed by immunoblotting with the indicated antibodies against DCAF8L1 and HA antibody. The results show that the home-made antibodies D8L1-NP and D8L1-1492, as well as the DCAF8L1 commercial antibody (D8L1-NB, DCAF8L1 antibody for Novus Biologicals, NBP1-93435) are specific for IP. Antibody CP is specific but with low efficiency for IP.

(C) DCAF8L1 antibody DL1-519 and D8L1-NP do not cross react with DCAF8 and DCAF8L2. 293T cells were transfected with Flag and HA tagged DCAF8 (FH-D8), DCAF8L1(FH-D8L1), or DCAF8L2 (FH-D8L2). Cells were harvested and lysed. Exogenous DCAF8s were then pulled down by anti-Flag M2 Agarose (Flag IP) and blotted with the indicated antibodies. Flag-HA-

DCAF8 may interact endogenous DCAF8, which could lead to increased DCAF8 signal than HA signal in Flag IP. D8L2 antibody was raised against N terminal DCAF8L1 and purified using peptide corresponding to 69-103aa of DCAF8L2.

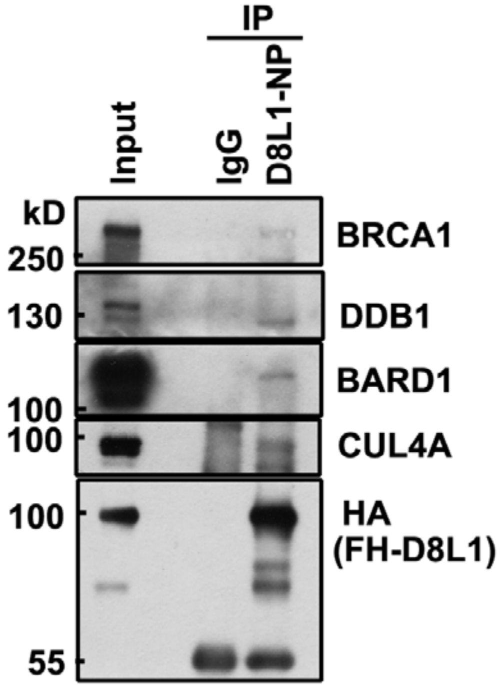

Fig.S2 DCAF8L1 interacts with BRCA1 and BARD1.

Exogenous DCAF8L1 interacts with endogenous BRCA1 and BARD1. Immunoprecipitation (IP) assays were performed on lysates from control or MCF10A cells stable expression Flag-HA–DCAF8L1 (FH-D8L1) using control IgG or antibody against DCAF8L1 (D8L1-NP). Immunoblots were analyzed with antibodies as indicated.

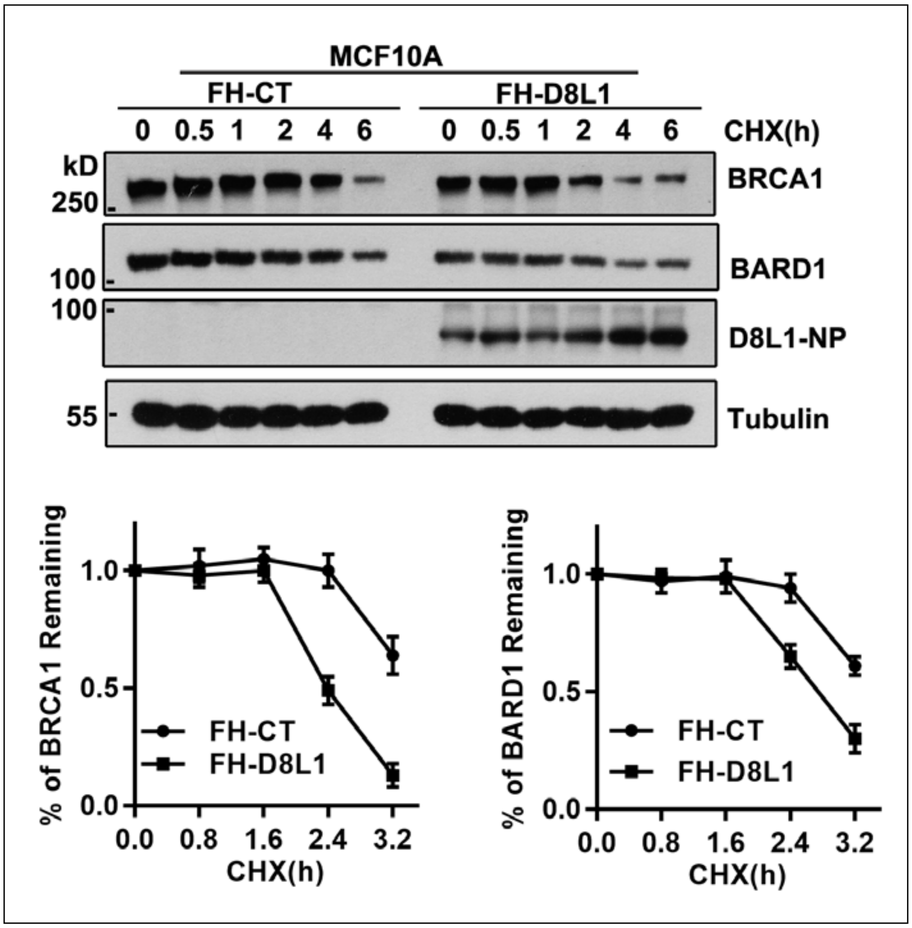

Fig. S3 Overexpression of DCAF8L1 accelerated degradation of BARD1 and BRCA1 proteins.

Forced expression of DCAF8L1 accelerated degradation of BRCA1 and BARD1 proteins in MCF10A cells. Cells were infected with TG006-FH or TG006-FH-D8L1 lentivirus. 72 h post-infection , cells were treated with CHX (100 μg/ml) to block de novo protein synthesis and then harvested at the indicated times points after CHX treatment and protein levels were analyzed by immunoblotting Quantification of the protein level of BARD1 or BRCA1 was plotted (bottom).

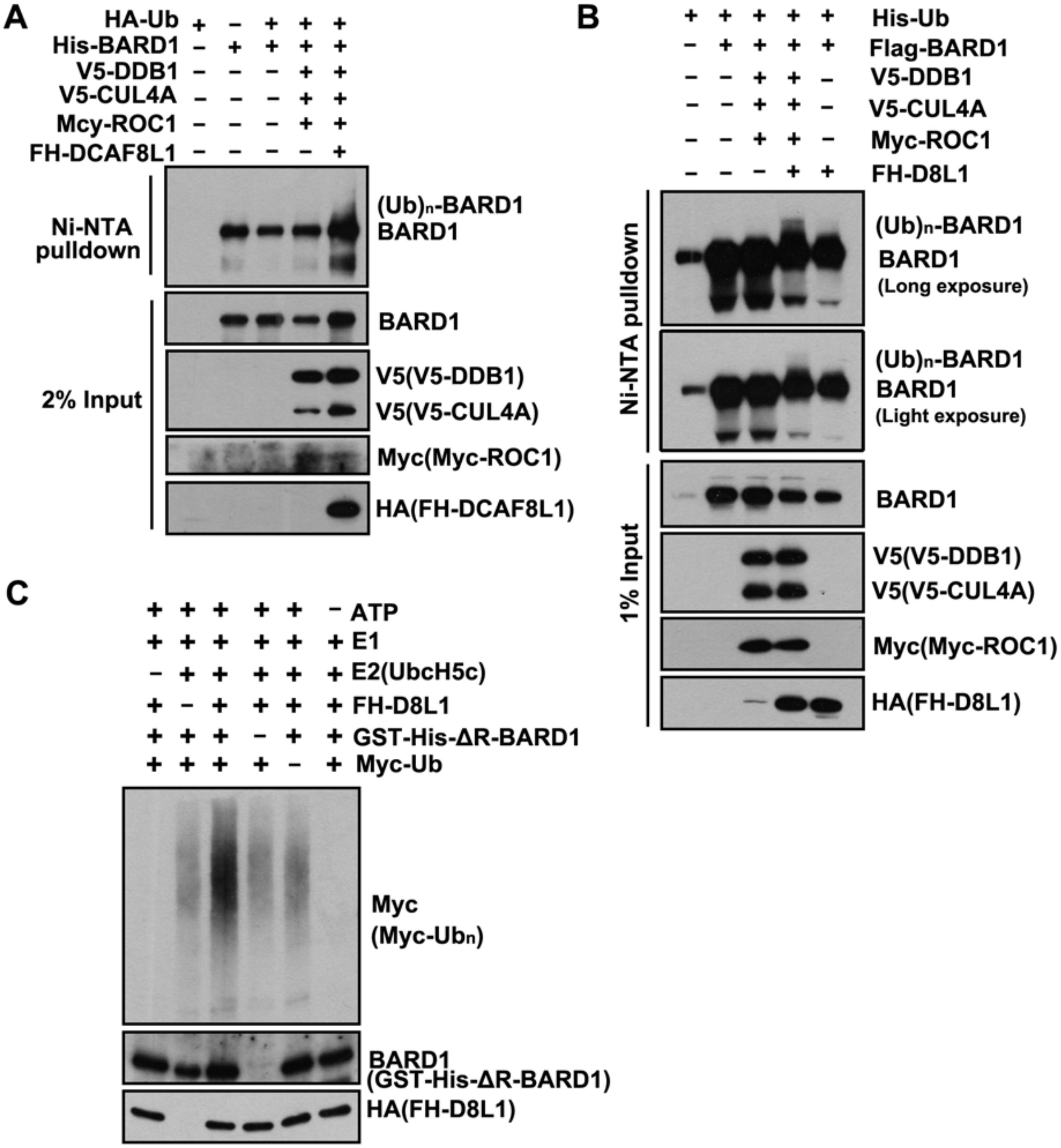

Fig.S4 CRL4^{D8L1} ubiquitinates BARD1 *in vivo* and *in vitro*.

(A, B) CRL4^{D8L1} ubiquitinates BARD1 *in vivo*. HEK293T cells transfected with the indicated plasmids, and His-BARD1 or His-Ub proteins were pulled down by Ni-NTA under denaturing condition. Immunoblotting was performed using the Ub or BARD1 antibodies.

(C) CRL4^{D8L1} ubiquitinates BARD1 *In vitro*. Recombinant GST-His-BARD1-119-777 (GST-His-ΔR-BARD1) protein were incubated with E1, E2 (UbcH5c), ATP regenerating buffer, recombinant Flag-HA-D8L1 protein complexes and Myc-ubiquitin in a 25 μl reaction volume for 1.5 h at 37℃. The reaction mixtures were analyzed by immunoblotting with Myc and BARD1 antibodies.

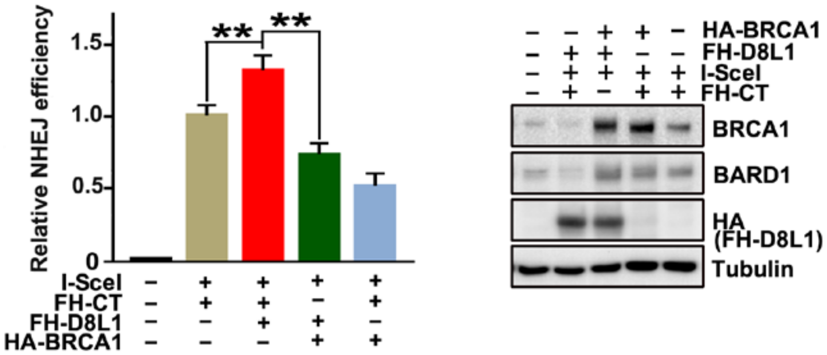

Fig.S5 Enhanced NHEJ in D8L1 overexpressing cells.
U2OS EJ5-GFP cells were transfected with the plasmids as indicated， GFP positive cells were analyzed by FACS and WB. NHEJ efficiency was expressed and normalized with I-SceI group as 1.

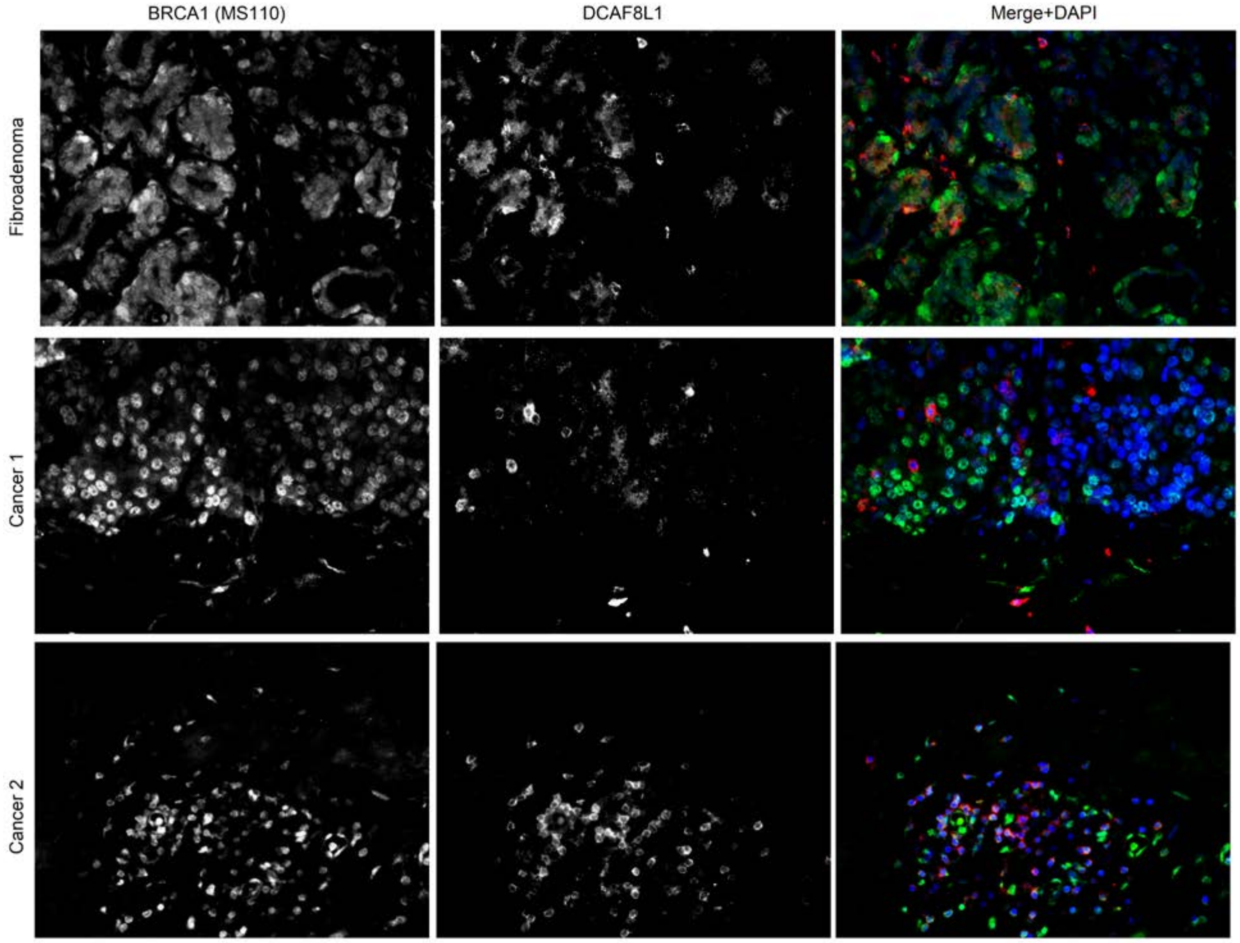

Fig.S6. DCAF8L1 overexpressing cells displayed loss or reduced BRCA1. Paraffin fixed breast fiboradenoma or cancer tissues were used for immunofluorescence co-staining with BRCA1 (MS110) and DCAF8L1 (Novus Biologicals). Green, BRCA1; Red, DCAF8L1; Blue, DAPI; Upper, breast fibroadenoma; middle and lower, breast cancer tissues.

2. Supplementary Tables

Table S1: Primer Sequences of Construction of Plasmid Used in This Study

Primers	Sequence (5’to 3’)	Used for
BamHI-hBARD1-1-F<br>XbaI-hBARD1-119-R	CGCGGATCCGCGCTGTCAGATTTGAAAGAA<br>GCTCTAGAGCTGTCAAGAGGAAGCAACTC	pFast-Bac-GST-BARD1-119-[illegible]
XhoI-hBRCA1-1<br>XhoI-hBRCA1-4	CCGCTCGAGGGATTTATCTGCTCTTCGCGTTG<br>CCGCTCGAGGTAGTGGCTGTGGGGGATCTG	pFast-Bac-Flag-BRCA1-His
pEGFP-L1-F<br>pEGFP-L1-R	ACAAGTCCGGCCGGATCAGATCTTCCGCTC<br>GAGCGGAAGATCTGATCCGGCCGGACTTGT	pEGFP-C2-D8L1
BamHI-DL1-519F<br>EcoRI-DL1-600R	CGGGATCCGGGTTAAAAGATGTGATTAAGA<br>CGGAATTC TCAGGATGGTATGCACTGCA	pGEX-6P-3-D8L1-519-600
BamHI-DL1-559F<br>EcoRI-DL1-600R	CGGGATCCGCTCATCAACCCGGCTGGAGA<br>CGGAATTCTCAGGATGGTATGCACTGCAC	pGEX-6P-3-D8L1-559-600
BamH1-DL1-14 F<br>EcoRI-DL1-92 R	CGGGATCCTTAGTGACTGAAAGCCTGTTC<br>CGGAATTCTCACGGGTAACCAAATAAACCTTC	pGEX-6P-3-D8L1-14-92
EcoRI-DL1-1<br>XhoI-DL1-2	CGGAATTCAATGTCCCACCAAGAGGGCAGC<br>CCGCTCGAGTCAGGATGGTATGCACTGCAC	pGEX-6P-3-D8L1-1-600
XbaI-DL1-1<br>KpnI-DL1-2	GCTCTAGAATGTCCCACCAAGAGGGCAGC<br>CGGGGTACCTCAGGATGGTATGCACTGCAC	pcDNA3.1-Flag-HA-D8L1
R317H-F<br>R317H-R<br>R365H-F<br>R365H-R	CACCATTGACCTCCATCAAGACCGGCCAG<br>CTGGCCGGTCTTGATGGAGGTCAATGGTG<br>GATTTATGACCAGCATAGAATTGATAAGA<br>TCTTATCAATTCTATGCTGGTCATAAATC	pcDNA3.1-Flag-HA-D8L1 Mt(R317,365H)
EcoRI-DL1-1<br>Xho1-DL1-2	CGGAATTCAATGTCCCACCAAGAGGGCAGC<br>CCGCTCGAGTCAGGATGGTATGCACTGCAC	pCMV-Tag2a-D8L1
Hind-BRCA1 F5-1<br>BamH1-BRCA1 F5-2	CCCAAGCTTGCCACCATGAAGAATGATATAACCAAA<br>CGGGATCCCCTGAGTGCCATAATCAGTACCAGGTAC	pCDNA4-Myc-His-ΔBRCA1

Table S2: key resources table

REAGENT or RESOURCE	SOURCE	IDENTIFIER
Antibodies		
Mouse monoclonal anti-BRCA1	Santa Cruz	sc-6954
Mouse monoclonal anti-BRCA1	Abcam	MS110
Rabbit polyclonal anti-D8L1-519	This paper	N/A
Rabbit polyclonal anti-D8L1-NP	This paper	N/A
Rabbit polyclonal anti DCAF8L1	Novus Biologicals	NBP1-93435
Mouse monoclonal anti-BARD1	Santa Cruz	sc-74559
Mouse monoclonal anti-Tubulin	Sigma	T6557
Mouse monoclonal anti-Myc	Cell Signaling Technology	2276S
Mouse monoclonal anti-Flag	Abcam	ab72469
Mouse monoclonal anti-HA	Abcam	ab18181
Rabbit polyclonal anti-V5	Abcam	ab9116
Mouse monoclonal anti-His	CST	2366
Mouse monoclonal anti-Ubiquitin	CST	3936
Rabbit polyclonal anti-CUL4A	Abcam	ab72548
Rabbit polyclonal anti-CUL4B	Novous Biologicals	NBP1-40587
Normal rabbit IgG	Santa Cruz	sc-2027
Normal mouse IgG	Santa Cruz	sc-2025
Mouse monoclonal anti-ROC1	Santa Cruz	sc-393640
Mouse monoclonal anti-OCT4	Santa Cruz	sc-5279

Mouse monoclonal anti-DDB1	Santa Cruz	sc-376860
Rabbit polyclonal anti-MAVS	CST	3993T
Rabbit polyclonal anti-Caspase-9	CST	9502T
Rabbit monoclonal anti-Cytokeratin 14	Abcam	ab181595
Mouse monoclonal anti-Cytokeratin 18	Santa Cruz	sc-6259
Bacterial and Virus Strains		
DH5α	Biomed	BS-3263
DH10Bac	Biomed	BC112-01
Stbl3	Biomed	BC108-01
Rosetta	Biomed	BC204-01
Biological Samples		
Human breast cancer and normal adjacent tissues	www.alenabio.com	BR1002a<br>BR20837
Chemicals, Peptides, and Recombinant Proteins		
Flag peptide	Sigma	F3290
HA peptide	Sigma	12149
MG-132	Sigma	M8699
Cyclohexane	Sigma	1810
Doxorubicin	Sigma	D1515
Olaparib	Sigma	SML1858
Etoposide	Sigma	E1383
Critical Commercial Assays		
GTVision TM III Detection System/Mo&Rb	Gene Tech	GK500705
Deposited Data		

Human reference genome NCBI	Genome Reference Consortium	https://www.ncbi.nlm.nih.gov/nuccore
Experimental Models: Cell Lines		
Human: Passage 40 H9 cells	ATCC	N/A
Human: Passage 6 HCC1954 cells	ATCC	N/A
Human: Passage 25 MCF10A cells	ATCC	N/A
Human: Passage 45 MCF10F cells	ATCC	N/A
Passage 3 Mouse embryonic fibroblast(MEF) cells	ATCC	N/A
Experimental Models: Organisms/Strains		
NOD/SCID	The Experimental Animals Center of Peking University	N/A
Oligonucleotides		
siD8L1-1: 5'- GGGAUGGUGGUUUCCUGAATT-3'	GenePharma	N/A
siD8L1-2: 5'- GCAUCAUAUUGCGAGAAUATT-3'	GenePharma	N/A
siD8L1-3’UTR: 5’-CCUAAGUACACUGGACUUUTT-3’	GenePharma	N/A
shD8L1-1: 5'-GGTGAAGGTTTATTTGGTTAC-3'	GenePharma	T5046
shD8L1-2: 5'-GGCAGAAGCCAGTACTGAACT-3'	GenePharma	T5047
shD8L1-3: 5'-GGAGCCAGACTCTCCTTATAA-3'	GenePharma	T5048

Table S3: D8L1 Immunostaining Pattern Scoring

Staining Pattern	Score
Intensity	
Negative	0
Weak	1
Moderate	2
Strong	3
Proportion of cells staining	
No positive cells	0
1%-30% positive cells	1
30%-70% positive cells	2
70%-100% positive cells	3
Total score	

Total score of 0, negative; 2~3, low expression +; 4~6, high expression ++
